# The need for maximal sterile barrier precaution in routine interventional coronary procedures; microbiology analysis

**DOI:** 10.1186/s40001-016-0239-4

**Published:** 2016-11-10

**Authors:** Avi Peretz, Fabio Kuzniec, Diab Ganem, Nabeeh Salman, Dahud Qarawani, Offer Amir

**Affiliations:** 1 Clinical Microbiology Laboratory, Baruch Padeh Medical Center, Tiberias, Israel; 2 Faculty of Medicine in the Galilee, Bar Ilan University, Zefat, Israel; 3 Cardio-Vascular Institute, Baruch Padeh Medical Center, Poriya, Tiberias, Israel

**Keywords:** Maximal sterile barrier precautions, Coronary catheterization laboratory, Infection transmission

## Abstract

**Background:**

Maximal sterile barrier precautions (MSBP) including head coverings and face masks are advocated for use in invasive procedures, including coronary interventions. The rationale for MSBP assumes it is an obligatory measure for infection prevention. However, in many coronary catheterization laboratories, head coverings/face masks are not used in daily practice. This study prospectively evaluated the potential hazards of not routinely using head coverings/face masks in routine coronary interventions.

**Methods:**

This is a prospective study of ambulatory patients in hospital care. A total of 110 successive elective patients undergoing cardiac catheterizations were recruited. Patients were catheterized by several interventional cardiologists who employed only routine infection control precautions without head coverings or face masks. For each patient, we took blood cultures and cultures from the tips of the coronary catheters and from the sterile saline water flush bowl. Cultures were handled and analyzed at our certified hospital microbiology laboratory.

**Results:**

In none of the cultures was a clinically significant bacterial growth isolated. No signs of infection were reported later by any of the study patients and there were no relevant subsequent admissions.

**Conclusion:**

Operating in the catheterization lab without head coverings/face masks was not associated with any bacterial infection in multiple blood and equipment cultures. Accordingly, we believe that the use of head coverings/face masks should not be an obligatory requirement and may be used at the interventional cardiologist’s discretion.

## Background

Maximal sterile barrier precautions (MSBP) including head coverings and face masks are advocated by published guidelines for use in invasive procedures related to intravascular catheter placement and replacement [1]. The rationale for MSBP is that it protects both the operator and the patient from infection transmission [2]. Accordingly, the use of MSBP, including the use of a cap, mask, sterile gown, sterile gloves, and a sterile full body drape, for intravascular catheter insertion and/or guide wire exchange was recommended as category IB [1, 3]. The original coronary catheterization laboratory (cath lab) procedure in the 1970s involved brachial artery cut-downs and was, therefore, considered an operation requiring complete sterile technique. In 2006, the Society for Cardiac Angiography and Interventions (SCAI) published infection control guidelines for the cath lab. These SCAI infection control guidelines indicated that for patient preparation, aseptic technique requires the use of cap, mask, sterile gown, sterile gloves, and large sterile sheet [4]. The cardiac cath lab has evolved since, but remains a complex environment in which implantable devices, closure devices, and other equipment must be used in a secure sterile fashion. In defining recent cath lab protocol, infection control issues generated much expert discussion as the Joint Commission considered procedures in the cath lab as a sterile procedure, rather than a clean one. Eventually, the 2012 American College of Cardiology Foundation/Society for Cardiovascular Angiography and Interventions expert consensus document on cardiac catheterization laboratory standards update noted that it is reasonable to wear hats and masks in the cath lab, but they are not mandated except for certain high-risk procedures—those involving insertion of devices, such as prosthetic valves and electrophysiology devices, and to close septal defects and patent foramen ovale. In these cases it was recommended that each laboratory should have a written protocol for increased sterile technique for highly infectious cases that should include caps, masks, double gloving, and protective eyewear [5]. In general, infectious complications in the cath lab are rare, ranging between 0.1 and 0.6% [6]. However, previous reports did suggest that infection transmission may be relevant for interventions done in the cath lab and that implementation of “full dressing” protocols decreases vascular catheter-related infection [7, 8]. In contrast, there is no evidence that rates increase without the use of hats and masks. This may explain the “daily practice” in which the many of interventional cardiologists in both the USA and Europe do not use head coverings and face masks. To clarify this debate and the struggle between regulatory instructions and daily practice, the purpose of the current study was to perform a systematic and thorough microbiological analysis in assessing the potential hazards of not using head coverings/face masks in percutaneous coronary interventions done routinely in the cath lab. To the best of our knowledge such a study in modern cath lab operating mode was not done in recent years.

## Methods

### Patients’ characteristics

A total of 110 successive ambulatory patients undergoing elective cardiac catheterizations were recruited. Patient characteristics are elaborated in Table 1.

**Table 1:** General characteristics of patients

Parameter	Descriptive statistics *n* = 110
Age, mean ± SD	57.9 ± 12.4
Man, *n* (%)	88 (80.0)
Diabetes mellitus, *n* (%)	42 (38.2)
Hypertension, *n* (%)	70 (63.6)
Permanent pacemaker, *n* (%)	6 (5.5)
PCI, *n* (%)	72 (65.5)

Patients were catheterized by several interventional cardiologists who employed only routine infection control precautions of standard hand washing, sterile gloves, and gowns without head coverings or face masks. All the procedures were diagnostic coronary angiograms and/or percutaneous coronary interventions. Exclusion criteria were: an index procedure for anti-arrhythmic device implantation or closure device, hemodialysis, previous catheters (temporal or permanent), active chemotherapy treatment, known immunosuppression status, or any recent history of febrile illness and/or antibiotic treatment. In each patient, at the end of the procedure in the cath lab, we took two sets of blood cultures (aerobic and anaerobic), one from a peripheral venous blood sample and one from the introducer arterial sheath inserted for the catheterization procedure. In addition, cultures were taken from the tips of the coronary procedure catheters and from the sterile saline water flush bowl. The study was approved by the IRB of the Poriya Medical Center, Tiberias, Israel. All patients signed a written informed consent prior to their participation.

### Microbiology workup

Cultures were handled and analyzed at our certified hospital microbiology laboratory. All blood cultures were incubated for a period of 7 days in BACTECTM FX (BD Diagnostics, Sparks, MD). This system is designed to detect microbial growth from blood specimens by measuring released CO_2_ produced through microorganisms’ metabolism. In case of a positive blood culture, a Gram stain would be performed for microorganism identification in accordance with its morphology and a culture would be made on a solid growth media for later microorganism identification by colony morphology characteristics. In addition, tips of catheters were sent to the clinical microbiology laboratory for microorganism colony identification and were seeded using sterile tweezers on blood agar plates with 5% sheep blood (BD Diagnostics, Sparks, MD). The water solution that was sent to the laboratory was spun in a centrifuge for 10 min at 3000 rpm and the sediment was seeded on blood agar growth media. All cultures were incubated 48 h at 37 °C. Each suspected bacterial growth was characterized by the VITEK 2 system (bioMérieux, Durham, NC), an automated system for bacterial identification.

## Results

Out of a total of 440 blood culture samples that were collected from 110 patients enrolled in the study, six were positive (1.3%). None of the patients enrolled in the study had more than one positive blood culture. All positive blood culture samples were aerobic in nature and presented coagulase-negative *Staphylococcus* (CoNS) growth. There was no bacterial growth in all the cultures collected from the ends of catheters and rinsing fluid. Further clinical follow-up of the patients demonstrated that none of the patients had developed any systemic signs of bacteremia or local infection proximate to area where the catheters were inserted.

## Discussion

The main finding of our current study is that not using full MSBP, specifically head coverings and face masks, is not associated with any clinically significant bacterial growth in either blood cultures, catheter tips, or sterile saline solution cultures. In addition, no clinical systemic or local infection was noted. To the best of our knowledge, this is the first study done in modern cath lab daily practice that focuses on a thorough microbiological assessment of such an important clinical question. Our current data support the daily practice of many interventional cardiologists in routine daily procedures done in the cath lab—not to use head covering and/or face mask as a measure for infection prevention. Our current study also supports current guidelines, which label these measures as a reasonable option rather than an obligatory one [5]. In this study, all cases of positive blood culture showed only in one bottle out of a set of bottles taken from the same area of each patient. This finding is in addition to coagulase-negative *Staphylococcus* growth, which is part of the normal skin flora, [9] probably due to wrong sample taking and lack of antiseptic technique use by the staff and not an infection that developed during the procedure [10–12]. Although the strict sterile techniques used in the operating room are not necessary for most cardiac cath lab procedures, Health Safety Guidelines and Usual Precaution Guidelines suggest that masks, an eye shield, and protective caps should be worn during cardiac catheterization as part of the sterile access field preservation [4]. This is why conflicts may occur between regulatory supervision bodies and the daily practice in many cath labs. We believe that our current analysis may clarify the safety of such practice and it will be decided by each interventional cardiologist’s discretion rather than by regulatory obligation. The main limitation of our study is the lack of a control arm. Indeed, it was our original intention to compare the results half way through the study of not using MSBP with a control arm of a routine use of MSBP. However, as we realized during the study itself that no clinically significant positive cultures emerged, we decided to continue with disuse of MSBP for the rest of the study. In summary, we demonstrated a thorough microbiological survey in 110 elective patients who had routine procedures in the cath lab, none of whom had any negative microbiological clinical consequences from the practice of not using head coverings or face masks routinely.

## Conclusion

Operating in the catheterization lab without head coverings/face masks was not associated with any bacterial infection in multiple blood and equipment cultures. Accordingly, we believe that the use of head coverings/face masks should not be an obligatory requirement and may be used at the interventional cardiologist’s discretion.
